# Supplementary material for: Identification and functional analysis of non-coding regulatory small RNA FenSr3 in Bacillus amyloliquefaciens LPB-18
Source: PeerJ. 2023 May 15;11:e15236. doi: 10.7717/peerj.15236 (PMC10194069; doi:10.7717/peerj.15236)
Supplement: Supplemental Information 4 [file peerj-11-15236-s004.zip › KO/CK-vs-T1_map/map04213.html]

KEGG PATHWAY: Longevity regulating pathway - multiple species - Reference pathway


|  |  |
| --- | --- |
| **Longevity regulating pathway - multiple species - Reference pathway** |  |

[
Pathway menu
| Organism menu
| Pathway entry
| Show description
| User data mapping
]

Aging is a complex process of accumulation of molecular, cellular, and organ damage, leading to loss of function and increased vulnerability to disease and death. Despite the complexity of aging, recent work has shown that dietary restriction (DR) and genetic down-regulation of nutrient-sensing pathways, namely IIS (insulin/insulin-like growth factor signalling) and TOR (target-of- rapamycin) can substantially increase healthy life span of laboratory model organisms. These nutrient signaling pathways are conserved in various organisms. In worms, flies, and mammals, DR reduces signalling through IIS/TOR pathways, deactivating the PI3K/Akt/TOR intracellular signalling cascade and consequently activating the antiaging FOXO family transcription factor(s). In yeast, the effects of DR on life- span extension are associated with reduced activities of the TOR/Sch9 and Ras/PKA pathways and require the serine-threonine kinase Rim15 and transcription factors Gis1 and Msn2/4. These transcription factors (FOXO, DAF-16, Gis1, and Msn2/4) transactivate genes involved in resistance to oxidative stress, energy metabolism, DNA damage repair, glucose metabolism, autophagy and protection of proteins by chaperones.

|  |  |  |
| --- | --- | --- |
| Reference pathway | 184% 150% 122% 100% 82% 67% 55% | 图片下载 |
